# Differential Role of Anterior Cingulate Cortical Glutamatergic Neurons in Pain-Related Aversion Learning and Nociceptive Behaviors in Male and Female Rats

**DOI:** 10.3389/fnbeh.2020.00139

**Published:** 2020-08-11

**Authors:** Sarah Jarrin, Abhay Pandit, Michelle Roche, David P. Finn

**Affiliations:** ^1^Pharmacology and Therapeutics, National University of Ireland Galway, Galway, Ireland; ^2^Centre for Pain Research, National University of Ireland Galway, Galway, Ireland; ^3^Galway Neuroscience Centre, National University of Ireland Galway, Galway, Ireland; ^4^Centre for Research in Medical Devices (CURAM), National University of Ireland Galway, Galway, Ireland; ^5^Physiology, School of Medicine, National University of Ireland Galway, Galway, Ireland

**Keywords:** anterior cingulate cortex, glutamate neurons, optogenetics, inflammatory pain, formalin, rat, c-Fos, conditioned place aversion

## Abstract

Pain is comprised of both sensory and affective components. The anterior cingulate cortex (ACC) is a key brain region involved in the emotional processing of pain. Specifically, glutamatergic transmission within the ACC has been shown to modulate pain-related aversion. In the present study, we use *in vivo* optogenetics to activate or silence, using channelrhodopsin (ChR2) and archaerhodopsin (ArchT) respectively, calmodulin-kinase IIα (CaMKIIα)-expressing excitatory glutamatergic neurons of the ACC during a formalin-induced conditioned place aversion (F-CPA) behavioral paradigm in both female and male adult Sprague-Dawley rats. Expression of c-Fos, a marker of neuronal activity, was assessed within the ACC using immunohistochemistry. Optogenetic inhibition of glutamatergic neurons of the ACC abolished F-CPA without affecting formalin-induced nociceptive behavior during conditioning. In male rats, optogenetic activation of ACC glutamatergic neurons decreased formalin-induced nociceptive behavior during conditioning without affecting F-CPA. Interestingly, the opposite effect was seen in females, where optogenetic activation of glutamatergic neurons of the ACC increased formalin-induced nociceptive behavior during conditioning. The abolition of F-CPA following optogenetic inhibition of glutamatergic neurons of the ACC was associated with a reduction in c-Fos immunoreactivity in the ACC in male rats, but not female rats. These results suggest that excitatory glutamatergic neurons of the ACC play differential and sex-dependent roles in the aversion learning and acute sensory components of pain.

## Introduction

Pain is comprised of both sensory-discriminative and affective-motivational components, which have distinct roles in the pain experience and can often modulate one another. Thus, it is not surprising that chronic pain and anxiety disorders are frequently co-morbid, with approximately 45% of chronic pain patients exhibiting a comorbid anxiety disorder (Lenze et al., 2001; McWilliams et al., 2003; Kessler et al., 2005; Korff et al., 2005; Roy-Byrne et al., 2008; Asmundson and Katz, 2009). Being able to target specifically the affective component of pain would be therapeutically beneficial for patients with chronic pain. In order to develop these improved treatments for chronic pain, there is a need for a better understanding of the neural circuitry involved in pain-related negative affect or aversion.

The anterior cingulate cortex (ACC) is a key brain region in the affective-motivational component of pain (Price, 2000). It has been found that lesion of the ACC reduces both formalin-induced conditioned place aversion (F-CPA) and visceral pain-induced CPA, pre-clinical behavioral paradigms used to investigate the affective component of pain, without affecting nociceptive responding (Johansen et al., 2001; Gao et al., 2004; Yan et al., 2012). Glutamatergic transmission and the expression of glutamatergic receptors in the ACC are increased in animal models of pain (Xu et al., 2008; Chen et al., 2014; Li W. et al., 2014; Yi et al., 2014; Hubbard et al., 2015; Liu et al., 2015), as well as clinically in patients with chronic pain conditions (Kameda et al., 2017; Lv et al., 2018). Studies have found that optogenetic activation of glutamatergic neurons in the ACC elicits mechanical allodynia in male mice while having no effect on nociceptive responding following an injection of complete Freund’s adjuvant (CFA). Conversely, optogenetic inhibition of glutamatergic neurons in the ACC has an antinociceptive effect of increasing paw withdrawal threshold in the mouse CFA-induced inflammatory pain model (Kang et al., 2015). It has been found that microinjections of the ionotropic glutamate receptor antagonist, kynurenic acid, into the ACC (Johansen and Fields, 2004) reduce aversion behavior in an F-CPA paradigm, while microinjection of the excitatory amino acid, homocysteic acid, into the ACC produces avoidance learning in the absence of a noxious stimulus in a CPA paradigm (Johansen and Fields, 2004). Thus, glutamatergic transmission within the ACC plays an important role in CPA.

Although chronic pain has a greater prevalence in women than in men (Fayaz et al., 2016), the vast majority of pre-clinical pain studies have only been conducted in males, with 79% of behavioral non-human animal pain experiments in papers published between 1996 and 2005 using male rodents only (Mogil and Chanda, 2005; Mogil, 2012). The inclusion of both sexes in pain studies is important because sex differences in pain have been observed, both in animal models and clinically (Berkley, 1997; Mogil and Bailey, 2010; Rhudy et al., 2010; Mogil, 2012; Sorge and Totsch, 2017). Due to the scarcity of pre-clinical pain studies performed in both males and females, little is known about sex differences in the role of glutamatergic neurons in the ACC in regulation of the sensory and affective components of pain.

In this study, we investigated the hypothesis that the glutamatergic neurons of the ACC have a facilitatory effect on pain-induced aversive behavior, possibly in a sex-dependent manner. The specific aims of the study were (1) to determine the role of glutamatergic neurons of the ACC in both formalin-induced nocifensive and aversive behaviors in female and male rats using optogenetic methodology and (2) to examine if behavioral changes are associated with alterations in expression of the marker of neuronal activity, c-Fos, in the ACC using fluorescent immunohistochemistry.

## Materials and Methods

### Animals

Experiments were carried out on adult male and female Sprague-Dawley rats (Table 1; Charles River, United Kingdom) maintained at a constant temperature (21 ± 2°C and relative humidity ranged from 36 to 49%) under standard lighting conditions (12:12 h light: dark, lights on from 08.00 to 20.00 h). All surgeries and behavioral trials and testing were carried out during the light phase between 08.00 and 19.00 h. Animals were group housed with three rats per cage until surgery after which they were singly housed. Cages were 42 cm × 26 cm × 13 cm and filled with 3Rs paper bedding (3Rs Lab, United Kingdom). A rectangle plastic insert was placed into the cage under the food hopper allowing animals to access food but preventing animals getting too close to the cage top which may result in damage to the optical fiber implants. Food (14% protein rodent diet, Harlan, United Kingdom) and water were available *ad libitum*. The experimental protocol was carried out following approval (Filing ID: 15/FEB/01) from the Animal Care and Research Ethics Committee, National University of Ireland, Galway, under license (project authorization number AE19125/PO28) from the Health Products Regulatory Authority in the Republic of Ireland and in accordance with EU Directives 86/609 and 2010/63 and were in accordance with ARRIVE guidelines from the National Centre for the Replacement Refinement and Reduction of Animals in Research (Kilkenny et al., 2010).

**TABLE 1:** Summary of experimental groups.

**Sex**	**Supplier**	**Weights**	**Age at surgery**	**Control (*n*)**	**ChR2 (*n*)**	**ArchT (*n*)**
Female	Charles River, United Kingdom	200–300 *g*	9–10 weeks	7	11	10
Male	Charles River, United Kingdom	400–500 *g*	9–10 weeks	9	8	10

### Virus Construction and Packaging

Recombinant adeno-associated viral (AAV) vectors were serotyped with AAV5 coat proteins and packaged by the viral vector core at the University of Pennsylvania, Philadelphia, PA, United States. Viral titer were 5 × 10^12^ particles/mL for AAV5.CAMKII.ChR2-mCherry.WPRE.hGH, AAV5.CAMKII. ArchT.eYFP.WPRE.hGH, and AAV5.CAMKII.mCherry. WPRE.hGH. Plasmids were provided by the Deisseroth lab, Stanford, United States.

### Stereotaxic Intracranial Viral Injections and Optical Fiber Implantation

Following delivery, rats were left to acclimatize to the animal unit for at least 4 days prior to surgery. They were then placed under isoflurane (2–3% in O_2_, 0.5 L/min) anesthesia and 0.5 μL of virus as specified above was bilaterally injected into the ACC (AP: + 1.5 mm; ML: ± 1.3 mm; DV: -1.3 mm at an angle of 12° toward the midline) at a rate of 0.5 μL/min. The microinjection needle was left in place for an additional 3 min prior to its removal. Rats were then bilaterally implanted with optical fibers (0.39 NA, 200 μm core multimode, Thorlabs, Germany) into the ACC (AP: + 1.5 mm; ML: ± 1.3 mm; DV: -1.0 mm at an angle of 12°toward the midline). Optical fiber implants were permanently fixed to the skull using stainless steel screws and glass ionomer dental cement (GC Europe, Kortrijk, Belgium). The non-steroidal anti-inflammatory drug, carprofen (2.5 mg/kg, s.c., Rimadyl, Pfizer, Kent, United Kingdom), was administered before the surgery to manage postoperative analgesia. To prevent postoperative infection, rats received a single daily dose of the antimicrobial agent enrofloxacin (5 mg/kg, s.c., Baytril, Bayer plc, Berkshire, United Kingdom) on the day of surgery and a subsequent 4 days.

### Formalin-Induced Conditioned Place Aversion

**FIGURE 1:**
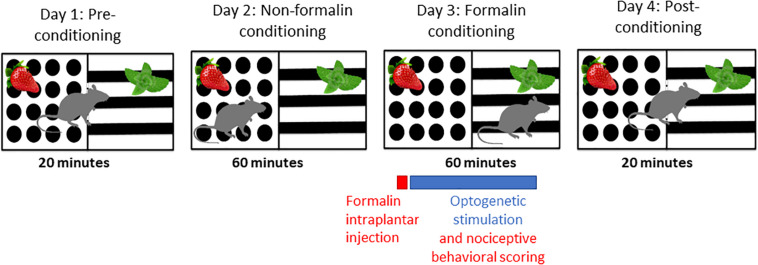
Schematic of F-CPA apparatus and behavioral testing timeline.

Animals were randomized to treatment groups and an experimenter blind to treatment carried out behavioral scoring. Behavioral testing was carried out 4 weeks after stereotaxic intracranial opsin encoding AAV injections. A two-chamber apparatus (each chamber 30 cm × 30 cm × 40 cm, l × w × h) with distinct odor (peppermint or strawberry) and visual (black and white balanced stripes or black dots on a white background) contexts was used for the F-CPA behavioral testing (Figure 1). The apparatus was tested and optimized in pilot experiments prior to the presented study so that there was no statistically significant difference in the time spent in the two chambers. The behavioral paradigm F-CPA combines the formalin test of tonic, persistent pain with the place-conditioning paradigm to measure pain-related aversion learning in rodents (Johansen et al., 2001). The test was run over 4 days: Day 1: pre-conditioning, Days 2 and 3: conditioning, and Day 4: post-conditioning. The pre-conditioning day consisted of a 20-min trial in which the rat was allowed free access between both chambers and the time spent in each chamber was recorded. The conditioning days consisted of a 60-min trial in which the rat was restricted to one of the chambers on each of the 2 days. On the second conditioning day (formalin conditioning), the preferred chamber from the pre-conditioning trial was paired with an intra-plantar injection of 50 μl formalin (2.5% in 0.89% saline, Sigma, Ireland) into the right hind-paw under brief isoflurane anesthesia (2% in O_2_; 0.5 L/min) as well as bilateral optogenetic stimulation at 10 Hz (15 ms pulse) with 2 s inter-pulse intervals for ChR2 groups and continuous stimulation for ArchT groups with a 465 nm LED light source (Plexon, United States) for the full 60-min conditioning trial. The behavior during the formalin-conditioning trial was recorded. The post-conditioning day consisted of a 20-min trial in which the rat was again allowed free access between the two chambers and the time in each chamber was recorded. The chambers were cleaned with warm soapy water and dried between each animal to remove odor cues. Male and female animals were tested on separate days, in the same apparatus, by the same experimenter, and under identical conditions.

### Behavioral Analysis

Behavioral trials were recorded and analyzed off-line using the EthoVisionXT11.5 software (Noldus, Netherlands) by a trained observer blind to the experimental conditions. Formalin-evoked nociceptive behavior was scored for the 60-min post formalin administration (day 3) according to the weighted composite pain scoring (CPS) technique (Watson et al., 1997). According to this method, pain behaviors are categorized as time spent elevating the formalin-injected paw above the floor without contact with any other surface (Pain 1), and holding, licking, biting, shaking or flinching the injected paw (Pain 2) to obtain a CPS [CPS = (Pain 1 + 2(Pain 2))/(duration of trial)]. For F-CPA, time spent in each chamber during the pre- and post-conditioning trials, as well distance moved and durations of rearing and grooming during the 60-min formalin conditioning trial were also assessed using EthoVisionXT11.5. The number of defecation pellets were counted during the 60-min formalin trial. F-CPA was calculated as duration spent in the formalin-paired chamber during the post-conditioning trial (day 4) minus the duration spent in that same chamber during the pre-conditioning trial (day 1). Therefore, a negative F-CPA score indicates an aversion to the formalin-paired chamber.

### Histology

Animals were euthanized and perfused immediately after completion of the post-conditioning trial on day 4 of testing. Brains were removed and post-fixed in 4% PFA in 0.1 M PBS for 24 h at 4°C before being transferred into 25% sucrose and 1% sodium azide in 0.1 M PBS. The ACC was later coronally sectioned (30 μm) using a freezing microtome and collected in 0.1 M PBS with 1% sodium azide (Sigma-Aldrich, Ireland). The positions of optical fiber tracts were noted during sectioning to locate and confirm placement in the ACC. Fluorophore-tagged opsin expression was confirmed for each brain by mounting sections onto gelatine-coated slides, cover slipping with VECTASHIELD Vibrance Antifade Mounting Medium with DAPI (Vector Labs, United Kingdom), and imaging them using an Olympus wide field inverted fluorescence microscope (Olympus, Tokyo).

### Fluorescent Immunohistochemistry

For confirmation of opsin expression and analysis of c-Fos immunoreactivity in the ACC, immunohistochemical staining was performed on free-floating sections. Sections were given 3 × 10 min washes in PBS, followed by an incubation for 1 h in 20% normal goat serum (Sigma Aldrich, Ireland) in PBS to block non-specific binding of the secondary antibody. Sections were then incubated in polyclonal rabbit anti-c-Fos antibody (Abcam, United Kingdom) at a concentration of 1:2,000 and rat anti-red fluorescent protein (RFP) antibody (Chromotek, Germany) at a concentration of 1:1,000 made up in PBS, 0.2% (v/v) Triton X, and 1% (w/v) normal goat serum for 24 h at room temperature under constant agitation. The sections were then given 3 × 10-min washes in PBS to remove the primary antibody and were then incubated for 3 h in 1:200 goat anti-rabbit secondary antibody in 10 μl/ml NGS in PB (Abcam, United Kingdom), tagged with either Alexa Fluor 488 for mCherry control and mCherry-tagged ChR2 sections or Alexa Fluor 594 for eYFP-tagged ArchT sections in order to distinguish the c-Fos labeling from the fluorophore-tagged opsin. Sections were kept in the dark and washed 3 × 10 min in PB and stored at 4°C until mounted onto gelatin-coated slides cover slipped with VECTASHIELD Vibrance Antifade Mounting Medium with DAPI (Vector Labs, United Kingdom).

Sections were imaged using an Olympus widefield inverted fluorescence microscope (Olympus, Tokyo). The number of c-Fos immunoreactive neurons within a 1 mm^2^ area in the ACC were counted for at least 5 sections per rat. The mean number of c-Fos expressing cells was then calculated for each rat that had at least 5 non-damaged sections for analysis followed by the overall group means. Counting was performed with the aid of NIMH Image J software (Bethesda, MD, United States).

### Statistical Analysis

IBM SPSS Statistics for Windows, version 26.0 (IBM Corp., Armonk, NY, United States) was used to perform two-way repeated measures analysis of variance (ANOVA) and GraphPad Prism statistical package (Graphpad Prism version 8.02 for Windows, GraphPad Software, La Jolla, CA, United States) was used to perform all other analyses, including two-way ANOVAs and *post hoc* pairwise comparisons. Normality and homogeneity of variance were assessed using Shapiro–Wilk and Brown–Forshythe test, respectively. Two-way repeated measures ANOVA was used to analyze CPS in the formalin test and two-way ANOVA was used to analyze F-CPA with sex and optogenetic modulation as factors. Immunohistochemistry results were analyzed with two-way ANOVA. Kruskal–Wallis test was used to analyze non-parametric data. *Post hoc* pairwise comparisons were made using Fisher’s LSD and corrected Dunn’s tests where appropriate. Data were considered significant when *p* < 0.05. Correlation analysis on c-Fos and F-CPA data was performed using Pearson’s correlation. Results are expressed as group means ± standard error of the mean (±SEM).

## Results

### Histological Verification of Implant Locations and Opsin Expression

**FIGURE 2:**
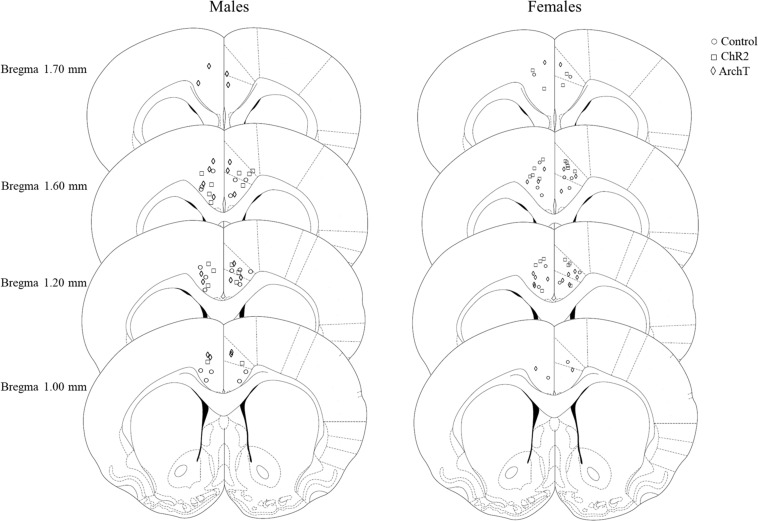
Schematic depicting sites of optical fiber implantation in the **left** and **right** ACC of male and female rats, adapted from Paxinos and Watson (2007).

After histological verification, 90% of males and 88% of females had implant tracts that were found to be within the borders of both the left and the right ACC. The remaining implants were placed in the corpus callosum, or outside the borders of the ACC. Only data from rats where optical fibers were accurately placed in both the left and the right ACC and that had opsin expression within the ACC have been included in the analysis (Figure 2).

### Formalin-Evoked Nociceptive Behavior

**FIGURE 3:**
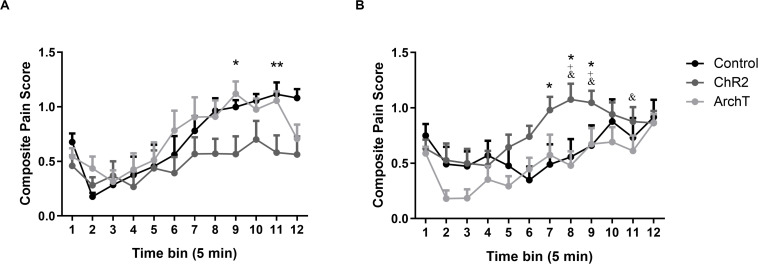
**(A)** Effects of optogenetic stimulation (ChR2) or inhibition (ArchT) of glutamatergic neurons in the ACC on formalin-evoked nociceptive behavior of male Sprague-Dawley rats. Data are mean ± SEM (*n* = 8–10 per group). **p < 0.01, *p < 0.05 ChR2 vs control. **(B)** Effects of optogenetic stimulation (ChR2) or inhibition (ArchT) of glutamatergic neurons in the ACC on formalin-evoked nociceptive behavior of female Sprague-Dawley rats. Data are mean ± SEM (*n* = 7–11 per group). *p < 0.05 ChR2 vs control. ^+^*p* < 0.05 ChR2 female vs ChR2 male, and ^&^*p* < 0.05 ArchT female vs ArchT male.

Intra-plantar administration of formalin into the right hind paw produced robust nociceptive behavior in both male and female SD rats as evidenced by the composite pain score. A two-way repeated measures ANOVA revealed significant effects of time [*F*_(__1_,_49__)_ = 13.83; *p* < 0.0001], sex × optogenetic modulation interaction [*F*_(__2_,_49__)_ = 5.56; *p* < 0.01], and time × sex × optogenetic modulation interaction [*F*_(__22_,_49__)_ = 1.83; *p* < 0.05] on formalin-evoked nociceptive behavior across the 60-min formalin trial but no effects of optogenetic modulation, sex, time × sex, or time × optogenetic modulation interactions (Figure 3).

*Post hoc* analysis revealed that optogenetic activation (ChR2) of glutamatergic neurons in the ACC significantly reduced (*p* < 0.05 or 0.01) formalin-evoked nociceptive behavior in male rats compared to controls at time bins 9 and 11 of the formalin trial (Figure 3A). By contrast, in female rats, optogenetic activation (ChR2) of glutamatergic neurons significantly increased (*p* < 0.05 or 0.01) formalin-evoked nociceptive behavior compared to controls at time bins 7–9 (Figure 3B). Moreover, formalin-evoked nociceptive behavior in the Female-ChR2 group was significantly higher than in Male-ChR2 counterparts at time bins 8 and 9 (*p* < 0.05). Optogenetic inhibition (ArchT) of glutamatergic neurons in the ACC had no significant effect on formalin-evoked nociceptive behavior in male or female rats, however, the Female-ArchT group exhibited significantly less formalin-evoked nociceptive behavior compared to Male-ArchT counterparts at time bins 8, 9, and 11 (*p* < 0.05; Figure 3).

### Effects of Optogenetic Modulation on General Locomotor Activity and Defecation in Formalin-Treated Rats

Two-way ANOVAs revealed that there were significant effects of sex on distance moved [*F*_(__1_,_49__)_ = 7.858; *p* < 0.01] and grooming [*F*_(__1_,_49__)_ = 15.82; *p* < 0.01] during the formalin trial, but no effects of optogenetic modulation of glutamatergic neurons in the ACC or sex × optogenetic modulation interaction (Table 2). Distance moved (*p* < 0.01) and duration of grooming (*p* < 0.001) were significantly greater in males than females regardless of optogenetic manipulation. Two-way ANOVAs revealed that there were no effects of optogenetic modulation or sex or their interaction on rearing or defecation (Table 2).

**TABLE 2:** Effects of optogenetic modulation of glutamatergic neurons in the ACC on general locomotor behavior and defecation during 60-min formalin conditioning trial in male and female rats.

	**Grooming (s)**	**Rearing (s)**	**Distance moved (cm)**	**Defecation (pellet number)**
Male-Control (*n* = 9)	207.340.2	25.410.1	3915719	1.60.5
Male-ChR2 (*n* = 8)	165.054.4	20.99.7	50371410	2.50.7
Male-ArchT (*n* = 10)	105.424.6	33.09.9	48841255	1.80.4
Male-Total (*n* = 27)	15723.5	26.95.6	4606650	1.90.3
Female-Control (*n* = 7)	57.215.7	19.713.4	2633289	2.70.8
Female-ChR2 (*n* = 11)	63.318.6	12.72.9	3003265	1.90.4
Female-ArchT (*n* = 10)	62.313.7	14.93.9	2475200	1.30.3
Female-Total (*n* = 28)	61.59.3***	15.13.3	2722147**	1.90.3

Data are mean ± SEM (*n* = 7–11 per group). ***p < 0.001, **p < 0.01 vs corresponding Male- Total, where Male-Total and Female-Total are the means ± SEM for all rats within each sex.

### Formalin-Induced Conditioned Place Aversion

**FIGURE 4:**
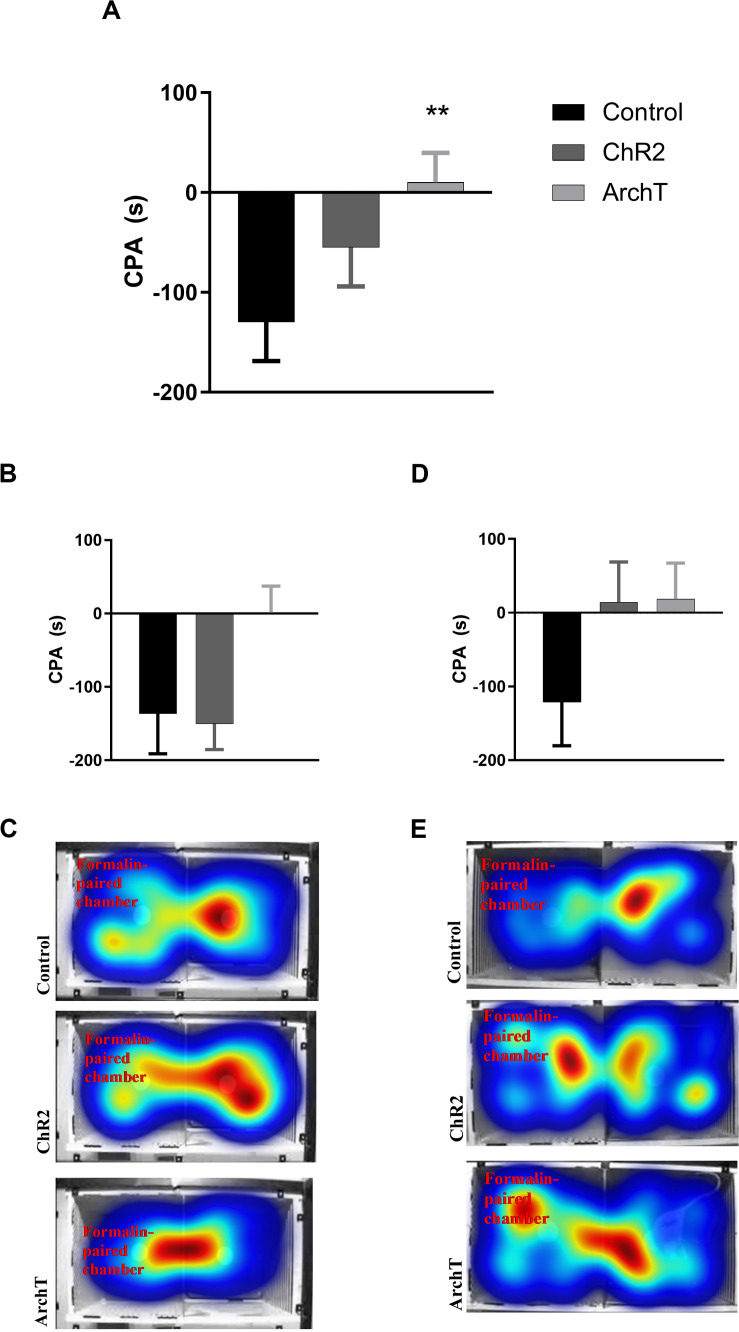
**(A)** Effects of optogenetic stimulation (ChR2) or inhibition (ArchT) of glutamatergic neurons in the ACC on formalin-induced conditioned place aversion (F-CPA) in combined male and female Sprague-Dawley rats. Data are mean ± SEM (*n* = 7–11 per group). **p < 0.01 vs control. **(B)** Effects of optogenetic stimulation (ChR2) or inhibition (ArchT) of glutamatergic neurons in the ACC on formalin-induced conditioned place aversion (F-CPA) in male Sprague-Dawley rats. Data are mean ± SEM (*n* = 8–10 per group). **(C)** Representative heat map images of post-conditioning trials for each treatment group in male rats. **(D)** Effects of optogenetic stimulation (ChR2) or inhibition (ArchT) of glutamatergic neurons in the ACC on formalin-induced conditioned place aversion (F-CPA) score of female Sprague-Dawley rats. Data are mean ± SEM (*n* = 7–11 per group). **(E)** Representative heat map images of post-conditioning trials for each treatment group in female rats.

Two-way ANOVA revealed an effect of optogenetic modulation of glutamatergic neurons in the ACC [*F*_(__2_,_49__)_ = 3.910; *p* = 0.03] on F-CPA behavior, but not of sex or sex × optogenetic modulation interaction (Figure 4). Optogenetic inhibition (ArchT), but not stimulation (ChR2), of ACC significantly reduced (*p* < 0.01) F-CPA behavior compared to control fluorophore-expressing rats, regardless of sex (Figure 4).

### c-Fos Immunoreactive Cells in the ACC

**FIGURE 5:**
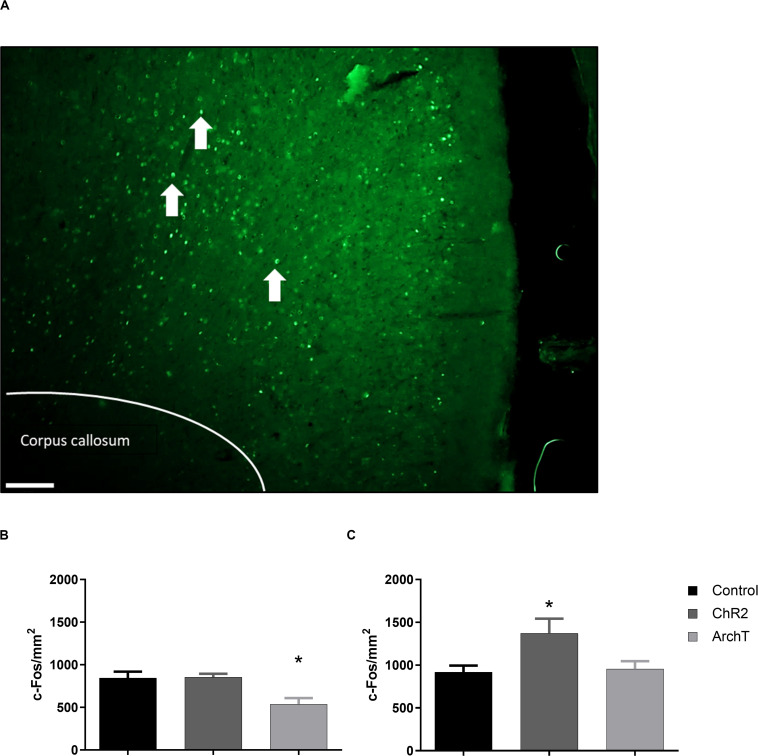
Effects of optogenetic stimulation (ChR2) or inhibition (ArchT) of glutamatergic neurons in the ACC on c-Fos positive cells in the ACC of SD rats. **(A)** Representative images of c-Fos (green) expression within the ACC (scale bar = 200 μm). White arrows denote representative c-Fos-positive staining. **(B)** Effects of optogenetic stimulation (ChR2) or inhibition (ArchT) of glutamatergic neurons in the ACC on c-Fos positive cells in the ACC of male SD rats. Data are mean ± SEM (*n* = 5–6 per group). *p < 0.05 vs. control. **(C)** Effects of optogenetic stimulation (ChR2) or inhibition (ArchT) of glutamatergic neurons in the ACC on percent of c-Fos positive cells in the ACC of female SD rats. Data are mean ± SEM (*n* = 4–6 per group). *p < 0.05 vs. control.

Two-way ANOVA revealed a significant effect of optogenetic modulation of glutamatergic neurons in the ACC during the day 3 formalin conditioning trial [*F*_(__2_,_24__)_ = 6.289; *p* < 0.01] and a significant effect of sex [*F*_(__1_,_24__)_ = 14.47; *p* < 0.01] on c-Fos-positive immunoreactive cells in the ACC after the day 4 post-conditioning trial (Figure 5). *Post hoc* analysis showed that optogenetic inhibition (ArchT) of glutamatergic neurons in the ACC during formalin conditioning significantly reduced the number of c-Fos-positive immunoreactive cells in the ACC of male rats compared to controls (Figure 5B; *p* < 0.05) and that by contrast, optogenetic activation (ChR2) significantly increased the number of c-Fos-positive immunoreactive cells in the ACC of females rats compared to controls (Figure 5C; *p* < 0.01). A correlation analysis was also performed between F-CPA score and the number of c-Fos-positive cells, for groups that exhibited significant differences in c-Fos expression (i.e., the control and ArchT groups for the male rats and the control and ChR2 groups for females). We found that there was a negative correlation between F-CPA score and c-Fos immunoreactivity in the ACC [*r*(9) = -0.59; *p* = 0.05] among the control and ArchT male rats, suggesting that greater formalin-induced aversive behavior was coupled with increased neuronal activity in the ACC in males. However, no correlation between F-CPA score and c-Fos was found in the control and ChR2 female groups [*r*(8) = 0.29; *p* = 0.41].

## Discussion

The results of the present study reveal differential effects of optogenetic modulation on formalin-evoked nociceptive behavior and F-CPA in male and female rats. We found that while optogenetic inhibition did not affect formalin-evoked nociceptive behavior in either sex, optogenetic activation of glutamatergic neurons of the ACC had a differential effect in males and females, reducing formalin-evoked nociceptive behavior in males during the second phase of the formalin trial and increasing formalin-evoked nociceptive behavior in females during this same stage of the trial. One mechanism suggested for the differences in pain observed between males and females is sex-dependent pathways for analgesia and hyperalgesia (Nemmani et al., 2004; Bryant et al., 2006; Bliss et al., 2016). Non-competitive antagonism of NMDA receptors potentiates morphine analgesia in male but not female mice (Nemmani et al., 2004), and attenuates morphine tolerance in male but not female mice (Bryant et al., 2006). Human studies have found differences in the functional connectivity between the subgenual ACC (sgACC) and various brain regions of the descending pain pathway in men and women (Wang et al., 2014; Monroe et al., 2017). For instance, women have greater functional connectivity between the sgACC and the periaqueductal gray (PAG) than men. It has been suggested that these projections from the ACC to the PAG may be involved in facilitating nociception through the RVM (Calejesan et al., 2000). If the functional connectivity of the ACC to the PAG is stronger in females compared to males, this difference could explain how optogenetic activation of glutamatergic neurons of the ACC increased formalin-evoked nociceptive behaviors in females and not in males.

Another possible mechanism underlying sex differences in pain is the influence of sex hormones (Martin, 2009). In particular, the affective component of pain has been found to be modulated by sex hormones. In female rats, pain-related aversive behavior is attenuated by ovariectomy (Chen et al., 2014; Li L.-H. et al., 2014; Hubbard et al., 2015). Inhibition of estrogen receptors or aromatase androstatrienedione in the ACC blocks F-CPA in both male and female rats, and exogenous estrogen elicits aversive behavior in the absence of a noxious stimulus in both sexes (Xiao et al., 2013). A limitation of the present study is the lack of estrous cycle stage assessment, however, we did not observe any effect of sex on F-CPA. It has been reported that estrogen enhances glutamatergic excitatory postsynaptic currents (EPSCs) in the ACC, suggesting that the mechanism by which estrogen modulates affective pain may be through modulation of glutamatergic transmission in the ACC (Xiao et al., 2013). Similarly, female rats in a high estrogen state exhibit increased pain behaviors and reduced excitatory amino acid transporter (EAAT) function in the ACC compared to rats in a low estrogen (Moloney et al., 2016). These findings may explain why we saw an increase in nociceptive behaviors with optogenetic activation (ChR2) of glutamatergic neurons in the ACC of female, but not male, rats.

Another key finding of the present study is that the inhibition of glutamatergic neurons of the ACC is sufficient to abolish formalin-induced aversion learning without reducing nociceptive behavior. Our results support and extend previous work which showed that administration of an NMDA receptor antagonist into the ACC abolished F-CPA in SD rats, while not affecting formalin-induced nociceptive behavior (Johansen and Fields, 2004; Lei et al., 2004a; Ren et al., 2006), suggesting that glutamatergic neurons in the ACC are active during pain states and are responsible for the aversive component of pain and that it is possible to dissociate this aversive component of pain from the sensory component. Previous work has also demonstrated that the NMDAR subunits NR2A and NR2B, as well as the NMDAR downstream ERK pathway, within the ACC, are necessary for pain-related aversion learning in rats (Cao et al., 2009; Li et al., 2009). Our immunohistochemical analysis revealed a reduction in c-Fos immunoreactive neurons in the ACC during the post-conditioning trial in male rats that received optogenetic inhibition of glutamatergic neurons in the ACC during the formalin conditioning trial, which was not seen in female rats that also received optogenetic inhibition. These results correspond to, and correlate with, the behavioral results observed, in that male rats that received optogenetic inhibition exhibited a reduction in formalin-evoked aversion behavior, which is in agreement with Lei et al. (2004b) who found that Fos immunoreactivity in the ACC is significantly increased after retrieval of formalin-induced conditioned place avoidance. By contrast, in female rats, optogenetic activation of glutamatergic neurons was associated with an increase in Fos immunoreactivity in the ACC. Interestingly, however, this increase was not coupled with the F-CPA behavior in females, as it was in males. There was no effect of optogenetic modulation on general locomotor activity, suggesting that the effect of optogenetic modulation on formalin-evoked nociceptive behavior was not a result of alterations in motor behavior. Female rats had lower levels of grooming and distance moved compared to males, but as there were no differences between control males and females for formalin-evoked nociceptive behavior, F-CPA or CPS, despite the difference in grooming, and since there were no effects of optogenetic manipulation on grooming, despite its effects on formalin-evoked nociceptive behavior, F-CPA or CPS, it is unlikely that sex differences in grooming have a bearing on the key findings and conclusions of this study.

A feature of the CPA behavioral paradigm is the necessary learning component, and thus it is possible that the effects of optogenetic modulation could result either from an effect on aversion behavior *per se* and/or an effect on general memory and learning. However, a previous study has found that optogenetic inhibition of glutamatergic neurons in the ACC of mice following induction of complete Freund’s adjuvant-induced arthritic pain induces place preference, suggesting that inhibition of these neurons does not inhibit memory acquisition or learning (Kang et al., 2017). It has also been found that optogenetic activation of glutamatergic neurons of the ACC in the absence of pain induces CPA to the stimulation-paired chamber (Tan et al., 2017).

The present study reveals, for the first time, sex differences in the role of glutamatergic neurons of the ACC in sensory and aversive components of pain. A particularly interesting result was the differential effect of activation of glutamatergic neurons of the ACC on formalin-evoked nociceptive behavior in males and females. The results of the study also support and extend the current understanding that inhibition of glutamatergic neurons of the ACC prevents aversion learning without affecting nociceptive behavior. Our findings identify glutamatergic neurons within the ACC as a potentially important substrate influencing sex differences in pain responding.

## Data Availability Statement

The raw data supporting the conclusions of this article will be made available by the authors, without undue reservation.

## Ethics Statement

The animal study was reviewed and approved by Animal Care and Research Ethics Committee, National University of Ireland Galway.

## Author Contributions

SJ and DF designed the experiments. SJ conducted the behavioral experiments and analysis of the c-Fos expression by immunohistochemistry, analyzed the data, and wrote the manuscript. DF, MR, and AP reviewed and edited the manuscript. All authors contributed to the article and approved the submitted version.

## Conflict of Interest

The authors declare that the research was conducted in the absence of any commercial or financial relationships that could be construed as a potential conflict of interest.
